# The Evil Effects of Some Dentifrices

**Published:** 1904-03-15

**Authors:** F. J. Turnbull

**Affiliations:** Scotland


### THE EVIL EFFECTS OF SOME DENTIFRICES.

BY F. J. .TURNBULL, SCOTLAND.

Some eighteen months ago my attention was attracted to certain pathological conditions of the gums and teeth in the following manner. I first noticed an inflamed condition of the gum margins, with tendency to decay on the labio-cervical surfaces of the incisor region. Secondly, it practically always happened with those people who paid particular attention to their teeth. Thirdly, it invariably occurred on the left side.

These thoughts gradually forced themselves upon my mind, and I found myself speculating on the cause. I first noticed, with some feelings of sadness, that my own left central incisor and the adjacent gum were beginning to exhibit similar symptoms, and had a series of cervical cavities. This made me think a little more seriously than before.

Various causes, including a hard tooth-brush and gouty diathesis, were considered. But the fact of it being always on the left side could not be got over. At last I came to the conclusion that the carbolic acid dentrifice which I was using was the culprit. The majority of people are right-handed, and after moistening their tooth-brushes they dip them into their powder, and the point of the brush naturally rests on the left incisor region first. This frequent application of a concentrated carbolic powder was, I considered, quite sufficient to cause an irritated condition of the mucous membranes. I therefore straightway put my theory into practice, and at first continued the use of a carbolic acid dentrifice, being careful, however, not to apply it to any particular place in a concentrated form. The result was most gratifying, and the gum became healthy and sound.

Since then I have discontinued a carbolic acid dentifrice and adopted another, which I will describe later. I have also had a considerable number of opportunities of noticing the results of my theory on patients, who have without exception benefited from the withdrawal of the carbolic, and the substitution of another dentifrice.

I must now detail to you the appearances as I have seen them.

The gum margin becomes red and inflamed, and slightly swollen. It then recedes from the labial surfaces of the teeth, becoming atrophied and anæmic in many cases. While these changes are taking place in the gums the teeth are suffering in consequence.

Having been denuded at the cervical margins of their natural covering of the gum, they are more susceptible to decay. At the same time they are subject to a more acid reaction, either from an acid secretion from the inflamed gum margin, or from a greater tendency to the collection of fermenting particles of *debris*. This causes a decalcification of the enamel and dentine, which results in a subsequent formation of cavities. My advice to patients is to change their dentifrice, as I have invariably found they are using carbolic acid powder; and secondly, to avoid the regular application of the brush to one particular spot at first; thirdly, I advise them to massage the gums with their forefinger, firmly in the case of inflamed and swollen gums, and gently in atrophic and anæmic conditions. When these particulars have been attended to, good results invariably follow, the gum resuming to a great extent its normal position.

This week I had the opportunity of seeing a patient whom I put under this treatment some five months ago, and I was very gratified with the improvement which had taken place in the interval.

In this case the lower incisor region had been also affected. The gums had receded considerably, and were thin and pale, although the patient was not otherwise anæmic. Now they are fuller and cover a greater portion of the root, being also of a better color. Until I put him under the treatment, the conditions were getting worse each time I saw him.

I fancy that with the most of us it is almost a daily question that we are asked, "What tooth-powder do you recommend?" Now in these days when every chemist not only sells dentifrices of his own manufacture, but those of countless manufacturers of toilet requisites, each of which are guaranteed to whiten and beautify the teeth, purify the breath, and strengthen the gums, it would be somewhat invidious to say which particular one was *the* best. Not only so, but our want of knowledgee of their exact ingredients does not even warrant us in saying which we *think* is best. Mark you, some may be good, probably quite so; but others are equally the reverse. A dentifrice which is claimed to whiten the teeth under *all* circumstances, either does so to the detriment of their structure or it does not fulfill all that is claimed for it. What are we to do then? Are we to waive responsibility in the matter and allow our patients to make a choice for themselves, or are we to do our duty and act as their guides and instructors? How much does the average patient know about the ingredients of a dentifrice, and how many, having a reliable dentifrice, know how to make a proper use of their tooth-brushes? I fancy if we made people give an exhibition of the way in which they use their tooth brushes, we could give most of them hints which would have beneficial results.

I know of course that this question requires delicate handling. It would be taken as an insult by most people to be told they did not clean their teeth. They all, or at least most of them, think they do. Some do so morning and night and after every meal. Others say life is too short, and they content themselves with a matutinal brushing To these I always say, "If you will do it only once a day then please do so at night," at the same time explaining my reasons for their doing so. Then there are a small minority who openly confess great carelessness in the use, or even entire neglect, of the tooth-brush altogether.

These people should all be spoken to as their requirements and habits necessitate. I must say that on more than one occasion when patients have asked me the question, "What should I *clean* my teeth with?" I have been very much tempted to answer "With a brush!" But it requires all our delicacy and tact to say all we would like to with sufficient emphasis, and at the same time not hurt their feelings. We should also remember that ours is not only a handicraft but a profession, and that advice and instruction constitute part of our daily work. Some people, a happy minority they are, require to pay little attention to their teeth. To these people a brush is all that is necessary. There are others who are less fortunate, however, some of whom have a viscid saliva with teeth practically never free from sordes. These unfortunate persons cannot pay too much attention to their teeth and gums in order to ward off decay.

Now it is obvious with these varied conditions we must vary our instructions to patients, and also explain why one dentifrice is not suitable for all. At the same time it is unnecessary to write out a prescription for every average person for daily use. One may have ready a supply of slips with a prescription either printed or written with cyclostyle, or if a chemist's shop be within easy reach one may request the proprietor to keep in stock a quantity. This he will be quite pleased to do, and it will be sold at a smaller cost to the patient than chemists would be prepared to dispense single prescriptions for. The following is the prescription which at present I recommend to my patients:

| | | |
|---|---|---|
| ℞ | Cretae praecipitatae - - - - - - | 1 oz. |
| | Magnes carbonatis - - - - - - - | 2 dr. |
| | Pulv. saponis cast. - - - - - - - | 1 dr. |
| | Saccharini - - - - - - - - - - - | 2 grs. |
| | Olei caryophyl - - - - - - - - - | 2 m. |
| | Menthol - - - - - - - - - - - - | 2 grs. |
| | Otto rosae - - - - - - - - - | 1 m. |
| | Carmine - - - - - - - - - - - - | q. s. |
| Sig. | To be used as a tooth powder as directed. | |

This powder I think is a good one for every-day use, as it has good cleansing, antiseptic, and alkaline properties, and at the same time no deleterious qualities.

Patients like the flavor and do not tire of it. Of course one will meet with people who say they detest cloves or menthol, or that the slightest frothing of soap in their mouths makes them sick. In these cases one must modify one's prescription, because it is a great thing to get people to use something they like.

Personally, I have used it myself for a considerable time and have not tired of it. The clove and menthol are not too pronounced. The saccharine imparts a sweet taste as well as being antiseptic.

The amount of soap does not make it froth too much, and yet is sufficient to assist in the removal of debris, and the magnesia aids the alkaline action.

One may add an astringent, such as tannin or powdered myrrh when necessary, or any of the various drugs to meet special requirements.

Before leaving the subject of dentifrices I would like to know if any gentlemen present have had any experience with patients who have used camphor dentifrices. I know in these days of fickle fashions it is difficult to meet any one who has used anything for long. But having been trained in my student days to avoid camphorated chalk as making the teeth chalky and brittle, I have always denounced it. I was doing so once to a patient who had particularly good teeth when she informed me she had never used anything else. This excellent condition of her teeth, however, may have existed in spite of the camphor.

In regard to gritty dentifrices I do not approve of adding cuttle-fish powder to them. I think it better that a pointed cane or stick with a very little fine pumice or cuttle-fish powder should be used when necessary and then only applied to the crevices actually requiring it.—*Dental Record.*

